# Is Self-Reported Physical Activity Participation Associated with Lower Health Services Utilization among Older Adults? Cross-Sectional Evidence from the Canadian Community Health Survey

**DOI:** 10.1155/2015/425354

**Published:** 2015-08-05

**Authors:** Koren L. Fisher, Elizabeth L. Harrison, Bruce A. Reeder, Nazmi Sari, Karen E. Chad

**Affiliations:** ^1^Department of Kinesiology, California State University, Fullerton 800 N. State College Boulevard, Fullerton, CA 92831, USA; ^2^Department of Kinesiology, University of Saskatchewan, 87 Campus Drive, Saskatoon, SK, Canada S7N 5B2; ^3^School of Physical Therapy, University of Saskatchewan, 1121 College Drive, Saskatoon, SK, Canada S7N 0W3; ^4^Department of Community Health and Epidemiology, University of Saskatchewan, Box 7, Health Science Building, 107 Wiggins Road, Saskatoon, SK, Canada S7N 5E5; ^5^Department of Economics, University of Saskatchewan, Arts 815, 9 Campus Drive, Saskatoon, SK, Canada S7N 5A5

## Abstract

*Purpose*. To examine relationships between leisure time physical activity (LTPA) and health services utilization (H) in a nationally representative sample of community-dwelling older adults. *Methods*. Cross-sectional data from 56,652 Canadian Community Health Survey respondents aged ≥ 50 years (48% M; 52% F; mean age 63.5 ± 10.2 years) were stratified into three age groups and analysed using multivariate generalized linear modeling techniques. Participants were classified according to PA level based on self-reported daily energy expenditure. Nonleisure PA (NLPA) was categorized into four levels ranging from mostly sitting to mostly lifting objects. *Results*. Active 50–65-year-old individuals were 27% less likely to report any GP consultations (OR_adj_ = 0.73;  *P* < 0.001) and had 8% fewer GP consultations annually (IRR_adj_ = 0.92;  *P* < 0.01) than their inactive peers. Active persons aged 65–79 years were 18% less likely than inactive respondents to have been hospitalized overnight in the previous year (OR_adj_ = 0.82,  *P* < 0.05). Higher levels of NLPA were significantly associated with lower levels of HSU, across all age groups. *Conclusion*. Nonleisure PA appeared to be a stronger predictor of all types of HSU, particularly in the two oldest age groups. Considering strategies that focus on reducing time spent in sedentary activities may have a positive impact on reducing the demand for health services.

## 1. Introduction

The importance of physical activity (PA) in reducing chronic disease and maintaining good health and functional independence has been well documented [1–5]. The health benefits of exercise, including enhanced cardiovascular functioning, improved glucose tolerance, and obesity reduction, are well known [1–3, 6]. Improvements in conditions such as osteoporosis, sarcopenia, and certain forms of cancer [1, 2, 4, 5, 7], positive changes in mental health, particularly related to depression and stress management, and improvements in cognitive ability, quality of life, and well-being [4, 5] have also been linked to increased PA levels. Although the importance of being physically active is widely acknowledged among the Canadian population, levels of physical activity remain low, particularly among older adults, with fewer than 15% attaining the recommended 150 minutes per week of moderate-vigorous PA [8, 9].

Physical inactivity among older adults is of particular concern in many industrialized countries, including Canada, because of the important societal implications associated with population aging [10]. By 2036 it is expected that 1 in 4 Canadians will be 65 years of age or older, the majority of whom will have at least one chronic condition [11–13]. Given that the average life expectancy at 65 years of age is now 21.5 years for women and 18.3 years for men, a significant proportion of the population will require ongoing, long-term medical care to manage their conditions [14]. There is great concern that the increasing chronic care needs of older adults will place considerable strain on the health care system, in terms of both its capacity to meet an increasing demand for services and its ability to sustain the current level of service provision in the face of increasing costs [15, 16]. Among policymakers and health providers alike, there is growing interest in the potential role of PA as a strategy to mitigate these challenges [10, 17, 18].

Physical inactivity has been shown to be positively associated with health service utilization and costs; however, the literature in this area is quite limited, particularly as it pertains to older adults [19, 20]. Recently published estimates of the economic burden of physical inactivity indicate that between 3.6% and 3.8% of total health care costs, or $6.8 billion, can be attributed to physical inactivity in Canada [21–23]. Studies examining the relationship between PA and health services utilization among older adults have shown mixed results. Woolcott et al. [24] compared “general health visits” (including general practitioner and specialist physicians as well as other health care providers) between active and inactive respondents in a nationally representative sample of 24,281 older adults aged 65 years and older. In this study, physically active seniors reported significantly fewer “general health visits” than their inactive counterparts (8.15 versus 11.76 visits/yr). In contrast, Plotnikoff et al. [25] found that PA was not significantly associated with either general practitioner (GP) or specialist visits in a sample of 2300 individuals with type 1 diabetes (T1D) or type 2 diabetes (T2D).

The results of studies of PA and hospital services utilization are also somewhat inconsistent. Sari [26] recently examined the association between PA and the demand for hospital services in adults who are 65 years of age and older and found leisure time PA (LTPA) to be inversely associated with hospital stays, concluding that even small increases in LTPA could translate into a decrease in hospital stays of 16% to 19% in inactive older adults. In a similar study, Woolcott et al. [24] reported that physically inactive older adults were 84% more likely to be hospitalized in the previous 12 months and spent, on average, more than three times the number of days in hospital compared to their active counterparts. In contrast to these studies, Plotnikoff et al. [25] failed to find a significant association between LTPA and the number of hospital visits in their sample of adults with T1D and T2D, after adjusting for other factors associated with health services utilization.

Although these studies are encouraging, there remain considerable gaps in our understanding of the relationship between physical activity and health services utilization, particularly among older adults [20, 27]. Along with the general lack of Canadian data, wide variations in study methodologies limit the extent to which we can draw conclusions from the literature [20]. The purpose of this study was to examine the relationship between PA and health services utilization, while controlling for a comprehensive set of observed covariates, in a nationally representative sample of community-dwelling adults aged 50 years and older in order to gain better insight into the relationship between physical activity and health services utilization.

## 2. Methods

This study involved a secondary analysis of data from Cycle 3.1 of the Canadian Community Health Survey (CCHS). The CCHS is a nationally representative cross-sectional health survey designed to provide information related to health determinants, health status, and health services utilization for the Canadian population aged 12 or older [28, 29]. Access to the confidential microdata files for Cycle 3.1 of the Canadian Community Health Survey (CCHS) was carried out through the Saskatchewan Research Data Centre at the University of Saskatchewan following an evaluation of the proposed research by the RDC-Access Granting Committee.

Data collection for CCHS Cycle 3.1 took place from January to December 2005 by means of computer-assisted personal and telephone interviews. In total 168,464 households were selected to participate. A response was obtained for 143,076 of the selected households, resulting in a household-level response rate of 84.9%. Of the 143,076 individuals selected (one person per household) to participate, valid interviews were conducted with 132,947 individuals yielding an individual-level response rate of 92.9%. When sample weights are applied, CCHS data represents approximately 98% of the Canadian population living in private and occupied dwellings in all provinces and territories [29, 30]. The present analysis was restricted to those respondents aged 50 years and older with nonmissing PA and health services utilization data, resulting in an unweighted sample of 56,652 adults.

### 2.1. Dependent Variables

Health services utilization was characterized as the use of general physician (GP) services, specialist physician (SP) services, and hospital services in the 12-month period prior to the survey. Both service contacts (services used versus services not used) and volume of service use were of interest because of the probable differences in the determinants of each type of utilization [31].

#### 2.1.1. Physician Services

Respondents were asked to report the number of consultations, including telephone consultations, with GP and specialist physicians in the 12-month period preceding the survey [32]. In addition to two continuous variables indicating volume of service use (one for each type of physician), two dichotomous variables (use versus nonuse) were constructed to indicate the incidence of contact with both types of physicians.

#### 2.1.2. Hospital Services

Two variables were used to describe the use of hospital services based on questions asking if the respondent had stayed overnight as a patient in a hospital and the total number of nights spent in hospital in the preceding 12 months: a dichotomous variable indicating if the respondent had been hospitalized in the previous year and, for those respondents reporting a hospitalization, a continuous variable indicating the total number of nights spent in hospital.

### 2.2. Independent Variables

The main independent variable of interest was self-reported LTPA over the 3 months prior to the survey interview. Respondents were asked about their participation in 21 specified physical activities, participation frequency, and average activity duration, using an adaptation of the Minnesota Leisure Time Physical Activity Questionnaire (MLTPAQ) [32]. An index variable was used to categorize respondents as active (>3.0 kilocalories per kilogram per day; KKD), moderately active (1.5 to 3.0 KKD), and inactive (<1.5 KKD), according to the average daily energy expenditure as determined by the reported frequency, duration, and metabolic cost associated with all the leisure time physical activity [32].

### 2.3. Control Variables

The control variables included in the analysis were chosen* a priori* based upon the Andersen-Newman model of health services utilization [33, 34]. Within this framework, individual determinants of health services utilization are categorized as “predisposing,” “enabling,” and “need” factors, all of which are thought to influence the decision to seek medical care.  Table 1 summarizes the predisposing, enabling, and need factors associated with health services utilization that were available in the CCHS Cycle 3.1 [29] and included as control variables in all statistical models. Selected environmental factors, including those related to the health system, the external environment, and the community, were also included, given their influence on health services utilization [35].

### 2.4. Statistical Analyses

All analyses were carried out at the Saskatchewan Research Data Centre using SPSS 19.0 (SPSS Inc., Chicago, IL) and STATA 10 (Statacorp LP, College Station, TX). To account for unequal probability of selection in the CCHS Cycle 3.1 due to the complex sampling design, sample weights were applied in all analyses in order to obtain population-based estimates [29]. Unless otherwise indicated, a significance level of *P* < 0.05 was applied.

In order to describe the characteristics of the study population, frequencies or means ± SD were determined as appropriate for all independent variables of interest. The sample was stratified on the basis of age and PA level into three age groups (50–64 years, 65–79 years, and 80 years and older) and three activity levels (active, moderately active, and inactive). The decision to stratify by age was made* a priori*, in recognition of the considerable heterogeneity within the demographic subgroups of the older adult population relative to PA, health, and health services utilization [15, 16, 26, 36].

Dependent variables were assessed separately for each age group. The distributions of all dependent variables were compared between PA groups using chi square and ANOVA for categorical and continuous variables, respectively. When the assumptions for these tests were not met, Fisher's exact test, Mann-Whitney *U* test, or the Kruskal Wallis test were used, as appropriate.

General linear modeling procedures were employed in order to assess the association between LTPA and each dependent variable. Multiple logistic regression models were used to obtain odds ratios (OR) describing the association between LTPA and the dichotomous variables indicating use or nonuse of physician services. Negative binomial (NB) regression modeling was used to obtain incident rate ratios (IRR) in order to assess the relationship between LTPA and the annual number of GP and specialist physician consultations [26, 37]. The association between LTPA and overnight hospitalizations and the total number of nights spent in hospital was assessed using ORs and IRRs obtained through multiple logistic regression and NB regression techniques, respectively. In all analyses, the reference group was the inactive category.

Bootstrap resampling procedures were used to produce corrected standard errors to calculate confidence intervals and to test for statistical significance. This technique is recommended for estimating sample variances in surveys with a large number of strata and multiple primary sampling units per stratum where exact design effects are not known [38]. A bootstrap macro specific to the CCHS Cycle 3.1 was provided by Statistics Canada.

## 3. Results

Key sociodemographic, health, and lifestyle characteristics of respondents are presented by age group and PA level in Table 2. In the 50 to 64 and 65 to 79 years age groups, the majority of the population was married and had completed high school. Most respondents in the oldest age group were not married and although the majority had completed high school, this age group was more evenly split across education levels. Across all age groups, less than 5% of the population self-identified as Aboriginal. The vast majority were born in Canada and lived in urban areas (>66% and ≥79%, resp.).

With regard to income, more than half of older adults under the age of 65 reported annual household incomes greater than $30,000; however fewer than 30% of respondents over the age of 65 reported annual incomes exceeding $30,000 per year. Approximately two-thirds of the population aged 50 to 64 years and 10% of those aged 65 to 79 years were employed. The majority of respondents under the age of 80 years reported living in a household with two or more people while close to half of those 80 years and older lived alone.

The vast majority (>90%) of respondents reported having a regular family doctor and this increased with increasing age. With the exception of inactive respondents in the two oldest age groups, more than 80% of respondents reported their general health to be excellent, very good, or good; more than 90% of respondents, regardless of age group, reported their mental health to be at the same level. At the same time, close to 80% of respondents under the age of 65 and 90% of those over the age 65 reported having at least one chronic condition. In each age group, the proportion of active older adults reporting no chronic conditions was higher than in the inactive and moderately active groups. The most prevalent chronic conditions were arthritis/rheumatologic conditions (24.5%–56%), hypertension (21%–49%), back problems (17%–26%), and cardio-/cerebrovascular conditions (6%–31%). The prevalence of most conditions was higher in older age groups and lower with increasing PA. One notable exception was in the case of mood/anxiety disorders, where the prevalence was lower in the older age groups compared to the youngest age group.

With regard to personal health practices, the vast majority of respondents (>75%) were nonsmokers or former smokers and most (>65%) reported that they did not consume alcohol. Except for inactive respondents in the oldest age group, at least 50% of respondents reported spending, on average, more than 1 hour daily walking to work and/or to complete errands while fewer than 5% reported cycling daily to do the same. The majority of respondents (40%–59%) reported standing or walking as their usual or typical daily activity. In each age group, the proportion of respondents reporting their usual activity as sitting was lower as the activity level increased.

Descriptive data for all health service utilization variables, stratified by age group and LTPA level, are presented in Table 3. In all age groups, the number of GP and specialist consultations differed significantly (*P* < 0.001) between each LTPA level. With the exception of specialist consultations in the oldest age groups, the moderately active group reported fewer GP and specialist consultations than the inactive group and the active group reported fewer GP and specialist consultations than either the moderately active or the inactive group. The majority of respondents reported having between 1 and 4 GP consultations and no specialist consultations in the previous year. In the youngest age group, between 16.5% and 22% of respondents had no visits to their GP in the previous year while, in the oldest age group, fewer than 13% had no visits. Across all age groups, between 29% and 36% of respondents reported at least one contact with a specialist physician in the previous 12 months.

Across all age groups, fewer than 20% of individuals had been hospitalized in the previous 12-month period. The proportion of respondents who had been hospitalized was highest in the inactive group and lowest in the active group regardless of age group. The number of nights spent in hospital differed significantly (*P* < 0.001) according to LTPA level, with the number of nights in hospital decreasing with increasing LTPA, across all age groups.

### 3.1. Regression Analyses

The results of regression analyses are presented separately for each age group.

#### 3.1.1. 50 to 64 Years Age Group

The results of the regression analyses pertaining to the 50 to 64 years age group are presented in Table 4. After adjusting for other factors related to health services utilization, active individuals in this youngest age group were 27% less likely than their inactive counterparts to have had contact with a GP in the 12-month study period (OR_adj_ = 0.73; *P* < 0.001). Being physically active was also associated with 8% fewer GP consultations over the 12-month study period (IRR = 0.92; *P* < 0.01). In contrast, moderately active and active 50- to 64-year-olds were as likely as their inactive counterparts to have at least one contact with a specialist physician (OR_adj_ = 0.85–0.94; *P* > 0.05) and were no more or less likely to be a high user of specialist services. Lastly, after adjusting for other determinants of hospital services (OR_adj_ = 0.77–0.93; *P* > 0.05) utilization, moderately active and active 50- to 64-year-olds were 8% and 3% less likely, respectively, to have had an overnight hospitalization in the previous 12 months; however, this was not statistically significant. Furthermore, LTPA was not significantly associated with the number of nights spent in hospital across activity level in this age group.

#### 3.1.2. 65 to 79 Years Age Group

The results of the regression analyses pertaining to the 65 to 79 years age group are presented in Table 5. In this age group, no significant associations were found between LTPA and the use of either GP or specialist physician services. While the adjusted analyses showed that moderately active or active individuals did have 4% fewer GP consultations than their inactive counterparts (IRR = 0.96), these groups were 3% (*P* < 0.05) and 13% (*P* < 0.05) more likely, respectively, to have had at least one contact with a specialist physician.

Lastly, after adjusting for other determinants of hospital services utilization, active 65- to 79-year-olds were 18% less likely to have had an overnight hospitalization in the previous 12 months (OR = 0.82, *P* < 0.05). Although PA was associated with between 3% and 7% fewer nights in hospital in this age group, this was not statistically significant.

#### 3.1.3. 80 Years and Older Age Group

The results of the regression analyses pertaining to the oldest age group are presented in Table 6. In this age group, no significant associations were found between LTPA and the use of either GP or specialist physician services. Moderately active or active individuals had 7–10% fewer GP consultations over the 12-month study period (*P* > 0.05). While LTPA was not significantly associated with the use of specialist physician services, moderately active and active individuals aged 80 years and older were 10% and 26% more likely, respectively, than their inactive counterparts to have had at least one contact with a specialist physician (*P* = 0.068 and *P* = 0.570, resp.) and active individuals also reported 6% more specialist visits (*P* > 0.05) over the 12-month study period.

The use of hospital services was not significantly associated with LTPA in the 80 years and older age group; however, moderately active and active individuals were 11% and 32% less likely, respectively, to report being hospitalized during the 12-month study period (*P* = 0.483 and *P* = 0.087, resp.). Among those reporting a hospitalization, active individuals spent approximately 20% more nights in hospital, although this was also not statistically significant (*P* = 0.401).

While not the primary variable of interest in this study, significant associations were found between typical daily activity and several of the dependent variables such that a brief presentation of these findings is warranted (see Table 7). Respondents were asked to choose the best description of their usual daily activities or work habits outside of their LTPA (sitting; mostly standing or walking; mostly lifting light or heavy loads) and, across all age groups, higher levels of usual activity were associated with lower health services utilization. In the 50- to 64-year-old age group, those reporting lifting light or heavy loads had significantly fewer GP consultations (IRR = 0.91; *P* < 0.05) and were 15% less likely to use specialist physician services (*P* < 0.05) compared to those reporting sitting as their typical daily activity. Among 65- to 79-year-olds, respondents reporting the highest level of usual activity were 23% significantly less likely to have contact with a GP physician (*P* < 0.05), had 13% fewer GP (*P* < 0.001) and 14% fewer specialist physician consultations (*P* = 0.065), were 32% less likely to be hospitalized overnight (*P* < 0.001), and spent 42% fewer nights in hospital than their sitting counterparts (*P* < 0.05). In the oldest age group, standing/walking and lifting light or heavy loads were associated with lower levels of GP and specialist physician service utilization compared to those whose typical activity was sitting (IRR = 0.66–0.89; *P* < 0.05). Lifting light or heavy loads was also associated with 48% fewer nights in hospital in this age group (*P* < 0.001).

## 4. Conclusion

The primary purpose of this study was to examine relationships between leisure time PA and health services utilization in a nationally (Canadian) representative sample of community-dwelling older adults. Rather than classifying all respondents as one homogeneous group, these relationships were explored separately for 3 age groups: 50 to 64 years, 65 to 79 years, and 80 years and older in order to add precision to the existing knowledge base [15]. The descriptive analysis showed that the use of health services generally increased with increasing age, with the exception of consultations with specialist physicians. Between 10% and 22% of respondents reported that they did not consult with a GP physician in the 12-month period, somewhat of a concerning finding from a health perspective given that it may mean that older adults are going without preventative health care or are having difficulty accessing necessary care. An alternative explanation may be that these individuals received health services from providers other than general practitioners, such as nurse practitioners, naturopathic physicians, chiropractors, and physiotherapists; however, this was not assessed in the present study.

The multivariate analyses showed that, in general, higher LTPA was associated with lower health services utilization; however, few of the associations were statistically significant. Leisure time PA was significantly associated with lower use of GP physician services in the 50 to 64 years age group, with active individuals 27% less likely to have contact with a GP and reporting 8% fewer GP consultations than their inactive counterparts in the 12-month study period. These findings are consistent with those of Woolcott et al. [24], as well as Wang et al. [39], who found that regular PA was associated with significantly lower outpatient health care costs in a group of Medicare retirees. Similarly, Mitchell et al. [40] also found physician visits to be inversely associated with physical fitness among 6,679 men aged 20–79 years. It is, however, important to note that the findings in this area are somewhat equivocal, with several studies reporting no significant association between LTPA and physician visits [27, 41, 42].

A significant association was also evident between LTPA and hospital services in the 65 to 79 years age group, where active individuals were 8% less likely to be hospitalized than their inactive counterparts. This partially supports the findings of recent studies by Woolcott et al. [24] and Sari [26] which found that LTPA was associated with a decreased likelihood of hospitalization and fewer nights spent in hospital among Canadians aged 65 years and older. While LTPA was mostly associated with fewer nights spent in hospital in the present study, these associations were not statistically significant. In the oldest age group, active respondents were actually more likely to report more nights in hospital. One explanation for the different findings may be related to the stratification of the sample of the present study. The studies by Woolcott et al. [24] and Sari [26] examined CCHS respondents aged 65 years and older as a single study population. There is considerable heterogeneity within the older adult population relative to PA, health, and health services utilization and it is possible that the stratification of the sample in the present study revealed differences in health services utilization that were obscured in studies which examined the population as a whole [15].

Although not statistically significant, the results pertaining to the use of specialist services revealed an interesting pattern. In the two oldest age groups, moderately active and active individuals were more likely than their inactive counterparts to have consulted a specialist in the previous 12 months. One possible explanation may be that moderately active and active older adults may be more health conscious and/or more health “literate” and therefore may seek referrals to specialists more frequently than inactive older adults [43]. The data related to specialist physician visits in the CCHS preclude an in-depth analysis of the physician specialty or the reasons underlying visits to specialists, both of which would provide important insights into the utilization of specialist physician services. However, despite its importance from a policy perspective, very few studies have examined the relationship of PA and specialist physician visits separate from visits to other physicians.

The lack of agreement between studies of PA and health services utilization may be due, in part, to considerable variation in sample populations, study design, and methods. There is no single “gold standard” measure of health services utilization and differences between studies in its operationalization make it difficult to form generalizations based on the available literature. Likewise, beyond the use of self-reported PA measures, there is very little consistency between studies in how PA is assessed. While most studies examining PA and health services utilization in older adults have used populations aged 65 years and older, the present study used a sample aged 50 years and older. There is significant heterogeneity in health status, PA participation, and health services utilization in the older adult population [15, 16, 36]. Also, there are a number of significant life transitions that typically occur after the age of 50, such as retirement and bereavement, which may have implications for health and health services utilization. Stratifying the data into smaller age groups coinciding with key transition periods and adjusting for age within each age group allows for a more precise analysis and comprehensive examination of the association between LTPA and health services utilization in this diverse population. For example, 50 to 65 years is the age range when many chronic conditions emerge and are diagnosed, hence the increased association with physician visits in this age group. In middle age group (65 to 79 years), chronic conditions may be worsening, resulting in stronger associations with hospitalizations. In both instances, LTPA may play an important role by either delaying the clinical manifestation of certain conditions or slowing progression of the disease process, thereby helping to delay or prevent this type of utilization in younger older adults.

Another notable difference between this analysis and previous studies was the inclusion of a wide-ranging set of control variables. A number of factors influence one's decision to seek medical care and the majority of earlier studies are lacking in their ability to account for other determinants of health services utilization, be they demographic and socioeconomic factors, physical and mental health status and medical comorbidities, or personal health practices such as smoking and drinking [26, 27, 37]. It is likely that physical activity affects health care utilization through its relationship with overall health [27]. By including a comprehensive set of health-related control we were also able to account for variations in health that may affect both the level of physical activity and healthcare utilization [26, 27, 37].

While LTPA was the primary focus of this study, respondents' typical daily activity outside of LTPA was also examined and appeared to be a stronger predictor of all types of health services utilization, particularly in the two oldest age groups. Even in the youngest age group, typical daily activity was significantly associated with the use of specialist services, where LTPA was not. One possible explanation may be that the typical daily activity variable may provide an indication of sedentary behavior, which is also associated with the development and chronic health conditions and poorer health status, independent of LTPA [44]. Among younger older adults still in the workforce, the amount of PA accrued during a typical day may exceed that accrued through LTPA due to the number of hours spent working; therefore this type of PA may be a more salient predictor of health services utilization. Another explanation may have to do with how respondents classified their own PA. Older adults typically participate in activities such as housework, gardening, and caregiving more frequently than other types of LTPA [45, 46]. Given that these types of PA were not specified in the CCHS instrument, respondents may have considered them as part of their usual daily activities. This highlights the importance of implementing measures of PA that are appropriate for older adults, given the types of PA typically reported in this population. It is possible that a more appropriate measure of LTPA may have revealed more significant associations with health services utilization. Lastly, among respondents aged 80 years and older, typical daily PA may be reflective of greater mobility and health status and, thus, be a stronger predictor of health services utilization.

Prior research in the area of PA and health services utilization has predominantly been focused on individuals in the workplace. This study is among a small few to examine the relationship between PA and health services utilization in community-dwelling older Canadians. Furthermore, the focus on both LTPA and typical daily PA is unique and provides new insights into the relationship between PA and health services utilization in the older adult population. Nonetheless, this study has certain limitations that should be considered when interpreting the results. The cross-sectional nature of the survey data precludes the inference of causal relationships and one cannot discount the possibility that reverse causality between the outcome measures and one or more independent variables is present. Furthermore, given the self-reported nature of the data, bias due to inaccurate recall or social desirability remains a possibility, particularly in the PA and health services data. Previous studies have shown that older adults tend to overreport contacts with GP physicians and underreport contacts with medical specialists, while recall of events such as hospitalizations appears to be more accurate, perhaps because these events are more highly salient and easily remembered [47, 48]. Likewise, there are issues with the use of self-reported measures of PA in an older population including vision and hearing impairments or disturbances to cognition and short- or long-term memory [49]. Additional problems may include the ability to accurately report activity intensity, because perceptions of what is “hard” activity or “light” activity depend on the tolerance and fitness level of the individual, both of which decline as a person ages [49].

Limitations owing to the CCHS instrument itself, while beyond our control, should also be pointed out. First, the discrepancy in recall periods between the health services utilization and LTPA variables may have made it more difficult to identify significant relationships; however, it would be considerably more difficult to accurately recall PA behaviors over a 12-month period compared to a lower frequency event such as health services utilization over the same period [27]. Furthermore, the measurement of LTPA in the CCHS may underestimate older adults' LTPA, particularly in the oldest age group, for at least two reasons: (1) the instrument does not specifically include more prevalent leisure time activities of older adults, such as housekeeping or caregiving and (2) the questionnaire may not be sensitive enough to detect the typically light and brief activity of elderly people [20, 49].

The above limitations notwithstanding, this study provides a significant contribution to a growing body of evidence suggesting that PA leads to lower health services utilization in community-dwelling older Canadians. Older adults are a very diverse group and this heterogeneity must be considered when examining health services utilization in this population. Although many of the estimates produced in the analyses were not statistically significant, they may have considerable relevance from a clinical perspective. For example, the results showed that moderately active and active respondents in the two oldest age groups were more likely to have at least 1 contact with a specialist and active individuals in the oldest age group appeared to have higher overall utilization of specialist physicians than their inactive or moderately active counterparts. Given that costs associated with specialist physician services are considerably higher than those associated with GP physician services, this finding warrants further exploration. Further studies of the patterns of health services utilization among sedentary, inactive, and active older adults would better clarify the potential role of PA as a strategy to decrease health services utilization and costs.* In addition, these findings suggest that both general and specialist physicians should be engaged in discussions related to physical activity given that older adults seek care from both groups of health professionals.*

In summary, it is possible that interventions aimed at increasing LTPA in this population may result in tangible reductions in health services utilization. The results also suggest that encouraging sedentary and inactive older adults, particularly those over age 65, to maintain or increase their overall daily activity, perhaps simply by reducing time spent in sedentary behaviors, may have an even greater impact on reducing the demand for health services. Given the wide variation in the literature with regard to study populations and methodologies, additional studies, with common outcome measures, appropriate and robust assessments of PA, and sedentary behavior, and adequate controls for confounders, are needed to obtain credible and accurate estimates of the effects of PA. Moreover, prospective longitudinal studies into the causal relationship between PA and health services utilization would provide important information on the potential impact would provide important insights about the potential impact of population-based strategies to increase PA participation among older adults on the health care system.

## Figures and Tables

**Table 1:** Predisposing, enabling, health need, personal health practices, environmental, and community control variables included in the multivariate analyses.

Control variables	Factors
Predisposing factors	Age; gender; marital status; education; ethnicity; immigration

Enabling factors	Annual household income; employment status; speaks English and/or French; has regular doctor; household size; dwelling size

Need factors	Self-rated general health; self-rated mental health; injury in the previous 12 months; limitations in ADLs; number of chronic conditions; chronic conditions^†^; BMI classification

Personal lifestyle factors	Smoking status; exposure to second hand smoke; alcohol consumption; walking/cycling for transportation; typical daily physical activity outside of leisure time

Environmental factors	Province; urban-rural residence

^†^Specified chronic conditions include hypertension, cardio-/cerebrovascular disease (heart disease, stroke); COPD (emphysema, chronic bronchitis); asthma; diabetes; cancer (currently have/ever had); neurological conditions (chronic fatigue, syndrome, migraines, Alzheimer's, other dementia, epilepsy); rheumatological conditions (fibromyalgia, arthritis/rheumatism); back problems; gastrointestinal conditions (intestinal/stomach ulcers; Crohn's disease/ulcerative colitis/irritable bowel syndrome/bowel incontinence); mood/anxiety disorders; other mental health conditions (schizophrenia, autism/other developmental disorder, eating disorder); conditions not otherwise listed.

**Table 2:** Population characteristics, stratified by age group and activity level (*N* = 56,652).

	Age group								
	50–64 yrs.			65–79 yrs.			80 yrs. and older		
Predisposing factors	(*n* = 29,914)			(*n* = 20,183)			(*n* = 6,555)		
	Inactive	Mod. active	Active	Inactive	Mod. active	Active	Inactive	Mod. active	Active
	(*n* = 15,393)	(*n* = 7,882)	(*n* = 6,639)	(*n* = 11,109)	(*n* = 5,027)	(*n* = 4,047)	(*n* = 4,772)	(*n* = 1,101)	(*n* = 682)
Age (mean ± SD)	56.2 ± 4.2	56.4 ± 4.3	56.5 ± 4.2	71.5 ± 4.3	70. 9 ± 4.2	70.6 ± 3.9	84.2 ± 3.6	83.2 ± 3.2	83.0 ± 2.9
Gender (%)									
Male	49.3	47.9	52.6	41.0	47.4	57.8	31.0	41.4	52.9
Female	50.7	52.1	47.4	59.0	52.6	42.2	69.0	58.6	47.1
Marital status (%)									
Married	76.0	78.9	79.5	64.8	69.6	72.5	37.0	41.1	53.5
Not married/missing	24.0	21.1	20.5	35.2	30.4	27.5	63.0	58.9	46.5
Education (%)									
Completed secondary	76.4	84.4	85.6	52.3	63.4	67.1	44.6	55.1	59.1
<Secondary	20.7	13.2	12.2	43.3	33.9	29.7	49.0	40.4	36.5
Missing	2.9	2.3	2.2	4.4	2.7	3.2	6.4	4.5	4.4
Household income (%)									
<$15,000	5.8	3.8	4.1	9.1	7.2	5.7	14.4	9.4	9.9
$15,000–$29,999	29.4	24.0	23.4	49.4	47.5	47.5	45.4	44.4	44.2
≥$30,000	50.0	59.4	59.7	19.8	27.5	27.1	12.0	20.4	23.0
Missing	14.7	12.7	12.8	21.7	17.8	19.7	28.3	25.8	22.9
Employment status (%)									
Not employed	30.4	32.7	37.3	85.6	89.2	87.7	100	100	100
Employed	67.2	65.0	60.7	11.7	9.1	10.1			
Missing	2.4	2.3	2.0	2.6	1.7	2.2			
Has regular family doctor (%)									
Yes	90.3	91.8	90.9	94.6	96.1	93.9	96.2	96.6	95.4
No/missing	9.7	8.2	9.1	5.4	3.9	6.1	3.8	3.4	4.6
Self-rated general health (%)									
Excellent/very good/good	80.8	88.8	91.9	69.8	83.6	88.0	63.5	81.2	82.5
Fair/poor/missing	19.2	11.2	8.1	30.2	16.4	12.0	36.5	18.8	17.5
Self-rated mental health (%)									
Excellent/very good/good	93.1	95.2	96.7	93.7	96.6	97.0	91.0	95.9	95.3
Fair/poor/missing	6.9	4.8	3.3	6.3	3.4	3.0	9.0	4.1	4.7
Number of chronic conditions (%)									
None/missing	20.3	20.0	23.3	10.1	10.7	14.1	7.7	10.9	13.1
1 condition	25.1	28.9	30.3	17.4	21.3	23.9	13.7	19.3	20.1
2 conditions	21.7	22.0	22.3	21.8	24.9	24.8	20.1	23.8	26.7
3 conditions	23.8	21.9	17.7	37.1	30.7	26.6	41.4	34.3	26.5
≥4 conditions	9.0	7.2	6.3	13.6	12.5	10.6	17.1	11.7	13.7
Typical daily PA (%)									
Usually sitting	27.5	21.6	17.0	30.4	12.3	7.4	48.1	21.5	11.0
Standing or walking	43.2	46.1	48.0	48.2	58.1	53.8	40.9	56.9	58.9
Lifting light/heavy loads	28.5	31.7	34.6	20.1	28.7	38.0	9.0	20.5	29.3
Missing	0.8	0.6	0.4	1.3	0.9	0.9	2.1	1.1	0.8

**Table 3:** Health services utilization, stratified by age group and PA level.

	50–64 yrs.			65–79 yrs.			80 yrs. and older		
	(*n* = 29,914)			(*n* = 20,183)			(*n* = 6,555)		
	Inactive	Mod. active	Active	Inactive	Mod. active	Active	Inactive	Mod. active	Active
	(*n* = 15,393)	(*n* = 7,882)	(*n* = 6,639)	(*n* = 11,109)	(*n* = 5,027)	(*n* = 4,047)	(*n* = 4,772)	(*n* = 1,101)	(*n* = 682)
GP consultations^*^									
Mean (SD)	3.5 (5.3)	3.1 (4.7)	2.7 (4.2)	4.2 (5.2)	3.6 (3.9)	3.3 (4.4)	5.4 (7.6)	4.4 (4.7)	3.9 (3.8)
None (%)	17.9	16.3	21.8	12.7	11.4	14.8	9.7	12.6	11.3
1 consultation (%)	20.2	23.4	23.8	14.4	16.9	20.8	11.0	14.4	16.4
2 to 4 consultations (%)	41.0	42.4	40.0	43.9	49.1	45.7	41.5	40.8	45.8
5 to 8 consultations (%)	11.8	11.3	9.2	16.1	13.5	11.7	19.0	17.3	16.0
9 or more consultations (%)	9.1	6.6	5.1	12.9	9.0	7.0	18.9	14.9	10.5
^†^Specialist consultations^*^									
Mean (SD)	1.1 (3.8)	1.0 (4.3)	0.8 (2.5)	1.0 (3.2)	0.8 (2.1)	0.8 (1.9)	0.9 (2.7)	0.8 (2.4)	0.8 (2.2)
None (%)	67.7	69.1	71.0	64.5	65.4	66.1	68.4	65.9	69.6
1 consultation (%)	13.9	13.8	14.5	14.9	16.4	15.8	12.3	15.8	16.8
2 consultations (%)	6.9	7.4	5.8	8.6	9.2	9.0	7.8	9.6	3.5
3 or more consultations (%)	11.5	9.7	8.7	12.0	9.0	9.1	11.4	8.6	10.1
Hospital admission in past year^*^ (%)									
Yes	7.8	6.1	5.7	14.1	9.9	8.5	19.7	14.4	10.5
No	92.2	93.9	94.3	85.9	90.1	91.5	80.3	85.6	89.5
^††^Total number of hospital nights^*^									
Mean (SD)	9.2 (21.2)	9.3 (23.4)	7.2 (16.8)	11.0 (21.2)	9.4 (20.1)	8.0 (17.6)	13.4 (25.8)	10.3 (16.1)	9.7 (12.6)

^†^Specialist consultations include all specialist physicians except eye specialists.

^††^Mean number of hospital nights for individuals reporting a hospital admission in previous 12 months.

^∗^Within each age group, PA groups were significantly different (*P* < 0.001) on all health service utilization variables.

**Table 4:** The association between LTPA and health services utilization in the 50 to 64 years age group (*N* = 29,914).

	50 to 64 years age group					
	Unadjusted			Adjusted		
	OR/IRR^a^	(95% C.I.)	Sig	OR/IRR	(95% C.I.)	Sig
GP services						
At least 1 GP contact						
Moderately active	1.12	(0.99–1.26)	0.061	1.01	(0.88–1.17)	0.854
Active	0.78	(0.70–0.88)	<0.001	0.73	(0.63–0.84)	<0.001
Number of GP consultations^a^						
Moderately active	0.88	(0.83–0.94)	<0.001	0.97	(0.92–1.03)	0.308
Active	0.78	(0.74–0.84)	<0.001	0.92	(0.87–0.97)	0.002
Specialist physician services^*^						
At least 1 specialist contact						
Moderately active	0.94	(0.86–1.02)	0.138	0.99	(0.89–1.09)	0.792
Active	0.85	(0.77–0.95)	0.002	0.99	(0.87–1.12)	0.882
Number of specialist consultations^a^						
Moderately active	0.93	(0.80–1.08)	0.339	1.01	(0.90–1.13)	0.858
Active	0.77	(0.65–0.90)	0.002	1.04	(0.90–1.21)	0.557
Hospital services^†^						
Overnight hospitalization						
Moderately active	0.77	(0.65–0.90)	0.001	0.92	(0.76–1.12)	0.416
Active	0.71	(0.58–0.86)	0.001	0.97	(0.77–1.23)	0.822
Number of nights in hospital^a^						
Moderately active	1.01	(0.69–1.49)	0.940	1.15	(0.94–1.43)	0.181
Active	0.78	(0.49–1.25)	0.303	1.03	(0.80–1.33)	0.816

^a^ The estimate is an incidence rate ratio (IRR).

Note: analyses adjusted for the following (reference category in italics): age; sex (*male*/female); marital status (married: *yes*/no); education (graduated secondary: *yes*/no); ethnicity (*non-Aboriginal*/Aboriginal); employment status (employed: *no*/yes); household size (*1*, 2, 3, or more people); dwelling size (*<3 bedrooms*, 3 bedrooms, >3 bedrooms); immigration status (*nonimmigrant*, immigrant); injury in previous 12 months (*no*/yes); limitation in ADLs (*no*/yes); smoking status (*never smoked*/former smoker/nonsmoker); exposed to 2nd hand smoke (*no*/yes); alcohol consumption (*<1 drink daily*/at least 1 drink daily); BMI (*<25.0* kg·m^2^/25.0–29.9 kg·m^2^/≥30 kg·m^2^ or greater); time spent walking to work or to run errands (*none*/<1 hour/≥1 hour); cycling to work or to run errands (*no*/yes); typical daily activity level (*usually sitting*/standing or walking/lifting light and/or heavy loads); annual household income (*<$15,000*; $15,000–$29,999; ≥$30,000; missing); province (*ON*, MB, AB, BC, SK, QC, NB, NS, PE, NL, YT/NT/NU); urban-rural classification (*urban*/rural); language (able to speak English and/or French: *yes*/no); has regular family doctor (*yes*/no); self-rated general health (*excellent/very good/good*; fair/poor); self-rated mental health (*excellent/very good/good*; fair/poor); diagnosed with hypertension, cardiovascular disease (including stroke), COPD, asthma, diabetes, cancer, neurological conditions, rheumatological conditions, back problems, gastrointestinal disorders, mood/anxiety disorders, or other chronic conditions (*no/*yes for each); number of chronic conditions (*none*/1 condition/2 conditions/3 conditions/4 or more conditions).

^*^Analyses of specialist services also adjusted for number of GP consultations.

^†^Analyses of hospital services also adjusted for specialist physician consultations (yes/*no*).

**Table 5:** The association between LTPA and health services utilization in the 65 to 79 years age group (*N* = 20,183).

	65 to 79 years age group					
	Unadjusted			Adjusted		
	OR/IRR^a^	(95% C.I.)	Sig	OR/IRR	(95% C.I.)	Sig
GP services						
At least 1 GP contact						
Moderately active	1.13	(0.97–1.32)	0.122	1.13	(0.95–1.34)	0.173
Active	0.84	(0.72–0.97)	0.016	0.99	(0.82–1.21)	0.959
Number of GP consultations^a^						
Moderately active	0.86	(0.81–0.90)	<0.001	0.96	(0.92–1.01)	0.123
Active	0.77	(0.72–0.82)	<0.001	0.96	(0.90–1.01)	0.122
Specialist physician services^*^						
At least 1 specialist contact						
Moderately active	0.96	(0.87–1.06)	0.427	1.03	(0.90–1.18)	0.658
Active	0.93	(0.83–1.04)	0.217	1.13	(0.98–1.31)	0.098
Number of specialist consultations^a^						
Moderately active	0.81	(0.73–0.91)	<0.001	1.02	(0.90–1.15)	0.780
Active	0.80	(0.71–0.90)	<0.001	1.02	(0.91–1.15)	0.709
Hospital services^†^						
Overnight hospitalization						
Moderately active	0.67	(0.57–0.78)	<0.001	0.90	(0.76–1.07)	0.229
Active	0.57	(0.49–0.66)	<0.001	0.82	(0.68–0.98)	0.032
Number of nights in hospital^a^						
Moderately active	0.85	(0.62–1.17)	0.328	0.97	(0.77–1.22)	0.795
Active	0.72	(0.52–1.01)	0.058	0.93	(0.71–1.22)	0.589

^a^The estimate is an incidence rate ratio (IRR).

Note: analyses adjusted for (reference category in italics) the following: age; sex (*male*/female); marital status (married: *yes*/no); education (graduated secondary: *yes*/no); ethnicity (*non-Aboriginal*/Aboriginal); employment status (employed: *no*/yes); household size (*1*, 2, 3, or more people); dwelling size (*<3 bedrooms*, 3 bedrooms, >3 bedrooms); immigration status (*nonimmigrant*, immigrant); injury in previous 12 months (*no*/yes); limitation in ADLs (*no*/yes); smoking status (*never smoked*/former smoker/nonsmoker); exposed to 2nd hand smoke (*no*/yes); alcohol consumption (*<1 drink daily*/at least 1 drink daily); BMI (*<25.0* kg·m^2^/25.0–29.9 kg·m^2^/≥30 kg·m^2^ or greater); time spent walking to work or to run errands (*none*/<1 hour/≥1 hour); cycling to work or to run errands (*no*/yes); typical daily activity level (*usually sitting*/standing or walking/lifting light and/or heavy loads); annual household income (*<$15,000*; $15,000–$29,999; ≥$30,000; missing); province (*ON*, MB, AB, BC, SK, QC, NB, NS, PE, NL, YT/NT/NU); urban-rural classification (*urban*/rural); language (able to speak English and/or French: *yes*/no); has regular family doctor (*yes*/no); self-rated general health (*excellent/very good/good*; fair/poor); self-rated mental health (*excellent/very good/good*; fair/poor); diagnosed with hypertension, cardiovascular disease (including stroke), COPD, asthma, diabetes, cancer, neurological conditions, rheumatological conditions, back problems, gastrointestinal disorders, mood/anxiety disorders, or other chronic conditions (*no/*yes for each); number of chronic conditions (*none*/1 condition/2 conditions/3 conditions/4 or more conditions).

^*^Analyses of specialist services also adjusted for number of GP consultations.

^†^Analyses of hospital services also adjusted for specialist physician consultations (yes/*no*).

**Table 6:** The association between LTPA and health services utilization in the 80 years and older age group (*N* = 6,555).

	80 years and older age group					
	Unadjusted			Adjusted^*^		
	OR/IRR^a^	(95% C.I.)	Sig	OR/IRR	(95% C.I.)	Sig
GP services						
At least 1 GP contact						
Moderately active	0.74	(0.55–1.01)	0.058	0.77	(0.52–1.15)	0.199
Active	0.84	(0.60–1.19)	0.324	1.26	(0.78–2.03)	0.340
Number of GP consultations^a^						
Moderately active	0.82	(0.74–0.92)	<0.001	0.93	(0.83–1.04)	0.225
Active	0.72	(0.65–0.81)	<0.001	0.90	(0.79–1.02)	0.113
Specialist physician services^*^						
At least 1 specialist contact						
Moderately active	1.12	(0.92–1.37)	0.263	1.26	(0.98–1.61)	0.068
Active	0.95	(0.74–1.21)	0.660	1.10	(0.80–1.50)	0.570
Number of specialist consultations^a^						
Moderately active	0.91	(0.69–1.20)	0.516	0.98	(0.80–1.21)	0.865
Active	0.91	(0.68–1.21)	0.506	1.06	(0.81–1.40)	0.661
Hospital services^†^						
Overnight hospitalization						
Moderately active	0.69	(0.53–0.89)	0.004	0.89	(0.65–1.22)	0.483
Active	0.48	(0.34–0.67)	<0.001	0.68	(0.43–1.06)	0.087
Number of nights in hospital^a^						
Moderately active	0.77	(0.55–1.08)	0.132	0.99	(0.69–1.43)	0.966
Active	0.72	(0.48–1.09)	0.119	1.19	(0.79–1.79)	0.401

^a^The estimate is an incidence rate ratio (IRR).

Note: analyses adjusted for the following (reference category in italics): age; sex (*male*/female); marital status (married: *yes*/no); education (graduated secondary: *yes*/no); ethnicity (*non-Aboriginal*/Aboriginal); employment status (employed: *no*/yes); household size (*1*, 2, 3, or more people); dwelling size (*<3 bedrooms*, 3 bedrooms, >3 bedrooms); immigration status (*nonimmigrant*, immigrant); injury in previous 12 months (*no*/yes); limitation in ADLs (*no*/yes); smoking status (*never smoked*/former smoker/nonsmoker); exposed to 2nd hand smoke (*no*/yes); alcohol consumption (*<1 drink daily*/at least 1 drink daily); BMI (*<25.0* kg·m^2^/25.0–29.9 kg·m^2^/≥30 kg·m^2^ or greater); time spent walking to work or to run errands (*none*/<1 hour/≥1 hour); cycling to work or to run errands (*no*/yes); typical daily activity level (*usually sitting*/standing or walking/lifting light and/or heavy loads); annual household income (*<$15,000*; $15,000–$29,999; ≥$30,000; missing); province (*ON*, MB, AB, BC, SK, QC, NB, NS, PE, NL, YT/NT/NU); urban-rural classification (*urban*/rural); language (able to speak English and/or French: *yes*/no); has regular family doctor (*yes*/no); self-rated general health (*excellent/very good/good*; fair/poor); self-rated mental health (*excellent/very good/good*; fair/poor); diagnosed with hypertension, cardiovascular disease (including stroke), COPD, asthma, diabetes, cancer, neurological conditions, rheumatological conditions, back problems, gastrointestinal disorders, mood/anxiety disorders, or other chronic conditions (*no/*yes for each); number of chronic conditions (*none*/1 condition/2 conditions/3 conditions/4 or more conditions).

^∗^Analyses of specialist services also adjusted for number of GP consultations.

^†^Analyses of hospital services also adjusted for specialist physician consultations (yes/*no*).

**Table 7:** The association between differing levels of typical daily activity^a^ and health services utilization, stratified by age.

	50 to 64 years			65 to 79 years			80 years and older		
	*n* = 29,914			*n* = 20,183			*n* = 6,555		
	OR/IRR^b^	95% CI	*P*	OR/IRR^b^	95% CI	*P*	OR/IRR^b^	95% CI	*P*
General physician services									
At least 1 contact with a GP									
Standing or walking	1.13	(0.96–1.32)	0.143	1.19	(0.96–1.47)	0.107	0.73	(0.50–1.07)	0.104
Lifting light/heavy loads	0.90	(0.76–1.06)	0.221	0.77	(0.61–0.98)	0.030	0.77	(0.46–1.28)	0.312
Number of GP consultations^b^									
Standing or walking	0.97	(0.92–1.02)	0.298	0.97	(0.92–1.02)	0.239	0.89	(0.81–0.99)	0.027
Lifting light/heavy loads	0.91	(0.86–0.97)	0.002	0.87	(0.82–0.93)	<0.001	0.84	(0.73–0.95)	0.006
Specialist physician services									
At least 1 contact with a specialist									
Standing or walking	0.95	(0.85–1.06)	0.366	0.88	(0.77–1.00)	0.048	0.85	(0.68–1.07)	0.169
Lifting light/heavy loads	0.85	(0.75–0.97)	0.020	0.86	(0.74–1.01)	0.065	0.72	(0.52–0.99)	0.041
Number of specialist consultations^b^									
Standing or walking	0.80	(0.70–0.93)	0.002	0.86	(0.75–0.98)	0.023	0.76	(0.63–0.92)	0.005
Lifting light/heavy loads	0.74	(0.64–0.86)	<0.001	0.82	(0.71–0.95)	0.006	0.66	(0.50–0.88)	0.005
Hospital services									
Overnight hospitalization									
Standing or walking	0.93	(0.77–1.12)	0.431	0.85	(0.72–1.01)	0.060	0.80	(0.62–1.03)	0.081
Lifting light/heavy loads	0.88	(0.70–1.09)	0.243	0.68	(0.56–0.84)	<0.001	0.92	(0.64–1.34)	0.678
Number of nights in hospital^b^									
Standing or walking	0.78	(0.64–0.94)	0.008	0.84	(0.68–1.03)	0.095	0.78	(0.59–1.02)	0.072
Lifting light/heavy loads	0.85	(0.67–1.09)	0.204	0.58	(0.45–0.76)	<0.001	0.52	(0.36–0.76)	0.001

^a^Typical daily activity is a 3-level categorical variable describing respondents' usual level of daily activity outside of LTPA. The reference group (not shown in table) is “usually sitting.”

^b^The estimate is an incidence rate ratio (IRR).

Note: adjusted for (reference category in italics) the following: age; sex (*male*/female); marital status (married: *yes*/no); education (graduated secondary: *yes*/no); ethnicity (*non-Aboriginal*/Aboriginal); employment status (employed: *no*/yes); household size (*1*, 2, 3, or more people); dwelling size *(<3 bedrooms*, 3 bedrooms, >3 bedrooms); immigration status (*nonimmigrant*, immigrant); injury in previous 12 months (*no*/yes); limitation in ADLs (*no*/yes); smoking status (*never smoked*/former smoker/nonsmoker); exposed to 2nd hand smoke (*no*/yes); alcohol consumption (*<1 drink daily*/at least 1 drink daily); BMI (*<25.0* kg·m^2^/25.0–29.9 kg·m^2^/≥30 kg·m^2^ or greater); time spent walking to work or to run errands (*none*/<1 hour/≥1 hour); cycling to work or to run errands (*no*/yes); typical daily activity level (*usually sitting*/standing or walking/lifting light and/or heavy loads); annual household income (*<$15,000*; $15,000–$29,999; ≥$30,000; missing); province (*ON*, MB, AB, BC, SK, QC, Other ); urban-rural classification (*urban*/rural); language (able to speak English and/or French: *yes*/no); has regular family doctor (*yes*/no); self-rated general health (*excellent/very good/good*; fair/poor); self-rated mental health (*excellent/very good/good*; fair/poor); diagnosed with hypertension, cardiovascular disease (including stroke), COPD, asthma, diabetes, cancer, neurological conditions, rheumatological conditions, back problems, gastrointestinal disorders, mood/anxiety disorders, or other chronic conditions (*no/*yes for each); number of chronic conditions (*none*/1 condition/2 conditions/3 conditions/4 or more conditions); number of GP consultations.
